# Families of Children with Intellectual and Developmental Disabilities: Variables Associated with Family Quality of Life

**DOI:** 10.3390/children11060734

**Published:** 2024-06-15

**Authors:** Ghaleb H. Alnahdi, Susanne Schwab

**Affiliations:** 1 Special Education Department, College of Education, Prince Sattam bin Abdulaziz University, Al-Kharj 11942, Saudi Arabia; g.alnahdi@psau.edu.sa; 2 Centre for Teacher Education, Department of Education, University of Vienna, 1010 Vienna, Austria; 3 Optentia Research Focus Area, North-West University, 1174 Hendrick Van Eck Boulevard, Vanderbijlpark 1900, South Africa

**Keywords:** family quality of life, intellectual disability, Beach Center FQOL scale, Saudi Arabia, mother, father, parents, siblings

## Abstract

Families of children with intellectual and developmental disabilities often face unique challenges that significantly impact their quality of life. Understanding the predictors of family quality of life (FQOL) is crucial for developing effective support systems and interventions. Aim: This study investigated the predictors that might influence the perception of families having a member with a disability regarding their quality of life (FQOL). Method: The sample consisted of 320 family members from the Riyadh region of Saudi Arabia. Results: The overall results showed that participants’ satisfaction with FQOL was at a moderate level. Further results indicated that variables associated with severity, type of disability, and the mother’s age and education were significant predictors of the FQOL. Conclusions: These results emphasize the importance of considering the variables that impact FQOL, such as the severity and type of disability, and mother’s related variables, when directing support to families with a member with a disability. The recommendations and limitations of the study were discussed.

## 1. Introduction

For complete inclusion, various barriers hampering social inclusion and giving people with disabilities a good quality of life must be dismantled. Even if similar barriers exist for people worldwide—for people with a disability, these barriers occur more often—they can have a strong impact on the lives of people with disabilities, their family members, and family functioning. According to the World Health Organization (WHO) and World Bank [1], for people with disabilities, achieving a high quality of life is linked to support. Focusing on the barriers to quality of life for people with disabilities is also promoted within the paradigm shift witnessed over the past few decades. In the past, a disability was defined using a medical model, but it is now defined using a social and human rights-based model (e.g., Council of Europe Disability [2]. In Saudi Arabia, according to the General Authority for Statistics [3], around 7.8% of people (aged above 5 years) are described as having a disability. Around 20% of people registered with a developmental disability have an intellectual disability in Saudi Arabia. Therefore, 7.8% of the population with a disability could be extended to a higher percentage of the country’s population considering that their family life will be influenced by members with a disability.

### 1.1. Family Quality of Life (FQOL)

A few decades ago, research demonstrated that families with a member having a disability face specific challenges, compared to families without any members with disabilities (e.g., see an overview of the literature review by Isa et al. [4]). For instance, in the United States, Zeng et al. [5] showed a rather moderate to low level of family quality of life (FQOL) for parents of children with autism spectrum disorders (ASD). Results from Meral et al. [6] in Turkey indicated that the FQOL of families with children having intellectual disabilities (ID) and ASD is slightly above the moderate level. In Croatia, Misura and Memisevic [7] showed that the FQOL of parents having children with intellectual disabilities was significantly lower compared to parents with typically developing children. Generally, interest in researching FQOL from the perspective of parents has been growing over the past few years (e.g., Ferrer et al. [8]). One reason may be that FQOL is an important family outcome [9], more specifically, a critical outcome of interventions, such as home-based support or family support [10].

Broadly, FQOL is seen as a complex multidimensional construct comprising several key domains [11]. As an extension of an individual’s quality of life, FQOL includes the entire family’s well-being and quality of life. Meanwhile, research focused extensively on families with children having disabilities has considered the entire family instead of single individuals [12]. An overview of the concept of quality of life in the field of intellectual disabilities can be found in Brown et al. [13,14]. Schalock et al. [15] stated that “quality of life is important for all people and should be thought of in the same way for all people—with and without disabilities”. Poston et al. [16] postulate that “as contrasted to individual quality of life, family quality of life addresses the impact of individual quality of life on the family—the interaction and reverberation of individual members as they produce the aggregate of family quality of life” [16] (p. 319). The authors addressed ten domains of FQOL—six individually oriented (advocacy, emotional well-being, health, physical environment, productivity, and social well-being) and four family-oriented domains (daily family life, family interaction, financial well-being, parenting). Vanderkerken et al. [11] also summarized that it is consensus that family interactions, physical/material well-being, parenting, and professional support are the core domains of FQOL.

As FQOL is related to negative physiological and psychological outcomes (Boehm et al. [17]), it is important to identify groups that are at risk of low FQOL and further predictors of FQOL.

### 1.2. Predictors of Family Quality of Life (FQOL)

In a recent study, Boehm and Carter [18] investigated factors linked with FQOL in a sample comprising parents of a family member with an intellectual disability. Their results show that several individual- and disability-related variables play a significant role in FQOL. For instance, FQOL was lower in families where a member had ASD. In Egypt, a study found that mothers of children with ASD expressed lower levels of FQOL compared to mothers who did not have a child with developmental disabilities [19]. In this context, Schmidt et al. [20] showed that families who had children with intellectual disabilities and ASD had a lower FQOL compared with families who had children with both intellectual and developmental disabilities. In addition, results from García-Grau et al. (2019) [21] indicated that having a child with ASD is a risk factor for low(er) FQOL. Moreover, the severity of the disability and the amount of support required correlated with FQOL [21]. The child’s age, however, did not correlate with FQOL in Boehm and Carter’s study [18], as in García-Grau et al. [21]. However, they showed that families with children from birth to 2 years had lower FQOL compared with families with children aged between 4 and 6 years. In addition, Giné et al. [22] show higher FQOL in families where children are aged above 18 years.

In addition, some variables for households/parents are associated with FQOL. Boehm and Carter [17] showed that parents with higher education and higher household incomes have more positive FQOL. Several other studies focused on the importance of parents’ educational attainment [20] and the amount of money available (e.g., family income, poverty) [22,23,24,25]. In addition, Giné et al. [22] demonstrated that marital status influences FQOL; specifically, their results indicated that families with married parents reported significantly higher FQOL compared to those with divorced or separated parents. It is still unclear if parents’ age plays a significant role, even though Meral et al. [6] showed that a mother’s age is associated with FQOL.

The previous literature suggests that the concept of FQOL has a cultural bias. According to (Schalock et al. [15], p. 461), “measuring quality of life reflects the unique blend of two meanings of quality: that which is commonly understood by human beings throughout the world and that which has become valued by individuals as they live their life within their unique environments”. The assumption that cultural/local aspects may influence FQOL is consistent with several studies. For instance, Boehm and Carter [17] indicated that the race/ethnicity of families having a member with disabilities plays a significant role in FQOL. This finding is relevant to our study as it underscores the importance of considering cultural and ethnic factors, which are highly relevant in the Saudi context where cultural norms and familial responsibilities are distinctively different from Western contexts. The authors showed that families with members who are only white had a higher FQOL compared to other families. Moreover, in some studies, religion and spirituality are linked with FQOL [16,17]. In Saudi Arabia, the approach to supporting individuals with disabilities and their families has seen a paradigm shift in recent years. Historically, care and support for persons with disabilities were primarily managed within the family unit, with a strong emphasis on the role of extended family members. This approach was consistent with the cultural and social norms of the region, where familial responsibility is highly valued.

The concept of quality of life has gained wide global attention over the past few years; nevertheless, only a few studies have dealt with this topic in the Arab world, specifically Saudi Arabia. It is only in the past five years that researchers in the Arab countries started to focus on this topic. In Jordan, Alawalmah [26] found that families rated their FQOL at a moderate level, and the type and severity of the disability were significant predictors of FQOL. Another study in Alegria found that a mother’s education plays a significant role in predicting FQOL [27].

### 1.3. Present Study

This study investigated the FQOL of families in Saudi Arabia that have a family member with a disability. To ensure easier understanding, the present study explains several predictors of FQOL. Even if some previous studies [17] investigated these predictors, this study adds something more meaningful, as it considers parents’ education (i.e., educational attainment of both parents) to derive specific results. In addition, the sampled study population of Saudi Arabia is expected to enhance understanding of the predictors in a different cultural context. This bi-directional understanding allows findings from Saudi Arabia to inform and be informed by results from other cultures, enriching the overall knowledge base on FQOL predictors globally.

Broadly, it is assumed that FQOL is rather moderate. We expect that several individual-related variables (type and severity of disability) and family/parent-related variables (monthly income, parents’ marital status, parents’ age, and education) influence FQOL. Comparing the variables of both mothers and fathers, we expect a higher impact on mothers’ variables because the existing literature has already documented the negative impact of mothers having a child with a disability (e.g., Bishop et al. [28]). The same is expected in Saudi Arabia as mothers are not only involved in caring for the family’s well-being but are also responsible for it. There are two main research questions in this study: (i) how do families having a family member with a disability perceive FQOL, and (ii) which variables have a significant impact on the FQOL of families having a member with a developmental disability?

## 2. Method

### 2.1. Sample

The study sample comprised 320 family members belonging to the Riyadh region of Saudi Arabia, and each family had at least one member with a developmental disability. Nearly half the sample (48%) comprised families having an individual with an intellectual disability and the rest were families having an individual with other disabilities (e.g., hearing impairment, visual impairment, autism, learning disabilities, multiple disabilities). Among the participants, 27% were mothers, 26% were fathers, brothers formed 16%, sisters comprised 11%, and the remainder (20%) were other relatives from an extended family such as an uncle or a grandfather (around 53% of participants were male and around 47% were female). More than half the sample has an individual with a moderate disability in their family (52%), 33% have an individual with a mild disability in their family, and 13% have an individual with a severe disability in their family. The mean of the participants’ age was 34 and SD = 13.3, while it was 12 years old for the member with a disability in the family with SD = 6.2. Participation in this study was on a voluntary basis.

### 2.2. Instrument

The Arabic version of the Beach Center Family Quality of Life Scale (BCFQOL-AR) is used in this study [29]. The BCFQOL-AR [30]) contains five subscales:

1. Family interaction (6 items): “My family solves problems together”.
2. Parenting (6 items): “Family members help children learn to be independent”.
3. Emotional well-being (4 items): “My family members have some time to pursue their own interests”.
4. Physical/material well-being (5 items): “My family gets dental care when needed”.
5. Disability-related support (4 items): “My family member with special needs has support to make progress at home”.

The Arabic version of the BCFQOL-AR scale uses five Likert options ranging from very dissatisfied (1) to very satisfied (5). As Alnahdi et al. [29] showed, the reliability of the overall scale (0.965) and subscales (0.854 to 0.946) was satisfied. The fit indices from the confirmatory factor analysis (CFA) indicate an acceptable fit, confirming the scale’s consistency with the original validation study. The reliability statistics for the overall scale and subscales were also detailed and supported, as shown in Appendix A and Appendix B.

### 2.3. Procedure

The study commenced with the identification and recruitment of participants from the Riyadh region of Saudi Arabia. Families with at least one member having an intellectual or developmental disability were targeted. Participants were informed about the study’s objectives, and voluntary consent was obtained. Data collection was conducted using the Arabic version of the Beach Center Family Quality of Life Scale. Participants completed the questionnaire, which included items on various aspects of family quality of life. The responses were collected and analyzed using SPSS, with a focus on descriptive statistics and hierarchical multiple regression analysis to identify key predictors of family quality of life. The procedures adhered to ethical guidelines to ensure confidentiality and respect for participants (approved by the Institutional Review Board committee and approved at Prince Sattam bin Abdulaziz University).

### 2.4. Analysis

To answer the research questions, statistical analysis was performed using SPSS 21. Descriptive statistics by demographic variables, descriptive statistics for the sample responses on all items, and hierarchical multiple regression analysis were used to examine the predictability of the independent variables. Before utilizing the regression results, we verified the necessary statistical assumptions as recommended [31,32]. We confirmed linearity, assessed the normal distribution of residuals, and evaluated the independence of errors using the Durbin–Watson statistic. Additionally, we checked for multicollinearity and verified this with variance inflation factor (VIF) values, ensuring the model was well-specified and valid.

## 3. Results

Table 1 shows the demographic statistics of the sample. The overall mean of 3.47 showed an average FQOL level (as the scale ranges from 1–5 the theoretical mean of the scale is 3). Families of individuals with intellectual disabilities (M = 3.32, SD = 0.99) expressed lower FQOL compared to families of individuals with other developmental disabilities (M = 3.60, SD = 0.93). Moreover, parents seem to rate FQOL lower compared to siblings and other relatives.

Table 2 shows the response percentage on each item of the FQOL scale. It is noticeable that four items with disability-related support items were the lowest with less than 50% of the sample strongly satisfied or satisfied with the support they received related to having a member with a disability. Participants expressed satisfaction with item 19 “My family gets medical care when needed”, with 73% strongly satisfied or satisfied, and item 17 “My family members have transportation to get to the places they need to be”, with 69% of the sample strongly satisfied or satisfied with this item.

### Predictors

In this section, we examine the predictability of variables that may influence FQOL. A multiple regression analysis was conducted (Table 3). Eight variables were entered into the model (type of disability, severity of the disability, monthly income, parents’ marital status, both parents’ age, and parents’ education). The regression model was significant (F (8, 252) = 6.73, *p* < 0.01), with an R^2^ of 0.238 and explained around 24% of the variances in FQOL. Three variables were significant at *p* < 0.01, including mother’s age, mother’s education, and severity of the disability. In addition, three other variables were significant at level *p* < 0.05 (type of disability, monthly income, and father’s age). However, mother’s age (younger age was associated with higher FQOL) and severity of disability (mild disability was associated with lower perceived FQOL) were the highest predictors associated with perceived FQOL. Father’s education and parents’ marital status were not significant predictors of FQOL.

## 4. Discussion

This study focused on the FQOL of Saudi Arabian families and its predictors. Broadly, the study findings show that families having a member with disabilities perceive a rather average level of FQOL. The mean of this sample was closer to the Turkish sample (M = 3.65 (Meral et al., 2013 [6])) than the American sample (M = 3.99 (Boehm et al., 2015 [17])). This is consistent with other studies [26] and confirms that family-centered approaches to support are meaningful when addressing the needs of persons with disabilities. More support is needed to understand the quality-of-life variables for families in Saudi Arabia as well as other countries in the Arab region. It is not that individuals with disabilities themselves have to deal with specific challenges, support is undoubtedly important for their family members. Interestingly, the study results show that siblings should also be the focus of research and practice, as they perceive a different level of FQOL compared to parents. This gap in research must be addressed as most studies only included parents’ perspectives and overlooked the view of siblings. For example, in Boehm et al.’s (2015) [17] study, around 87% of the sample were only mothers and only 19 participants did not identify themselves as parents of an individual with a disability. Moreover, in their study limitations, the authors stated that “fathers and siblings may hold very different perspectives on the quality of life experienced by their family” (p. 407). Moyson and Roeyers [33] also pointed out that siblings experience a unique type of FQOL.

Regarding the role of motherhood, our study highlights several significant findings. Younger mothers tend to manage the challenges associated with having a child with disabilities better, possibly due to their higher energy levels and more adaptable parenting styles. However, a critical and culturally relevant finding was that lower levels of maternal education were associated with higher FQOL. This counterintuitive result might be explained by the expectation levels; higher-educated mothers may have higher expectations for their children and family life, making it more challenging to cope with the realities of having a child with disabilities.

Regarding the role of motherhood, our study highlights several significant findings. Younger mothers tend to manage the challenges associated with having a child with disabilities with better satisfaction, possibly due to their higher energy levels and more adaptable parenting styles. However, a critical and culturally relevant finding was that lower levels of maternal education were associated with higher satisfaction with FQOL. This result might be explained by the expectation levels; higher-educated mothers may have higher expectations for their children and family life, making it more challenging to cope with the realities of having a child with disabilities. Moreover, the societal role of mothers in Saudi Arabia, and many other Arab cultures, places a significant amount of caregiving responsibility on them. This societal attitude could underscore the importance of providing emotional and social support to mothers, not just focusing on the physical and material aspects.

According to the family members with disabilities, the results of this study show evidence that the severity of the disability is linked to FQOL. It is not surprising that more severe disabilities are causing lower FQOL. Specifically, having a family member with intellectual disability continues to remain a huge challenge.

Another interesting result is the high impact of mothers’ age. It seems that younger mothers can deal with challenges related to their children with disabilities. This is particularly relevant in Saudi Arabia, where younger mothers might be more adaptable to changing social and family dynamics. In addition, the mother’s education also played a significant role. Lower levels of maternal education were associated with higher FQOL, which might be due to different expectations and societal pressures faced by educated mothers in Saudi Arabia. However, contrary to previous literature [22,34], the lower a mother’s education, the higher the FQOL. This can be explained by the fact that the mother’s higher education will be reflected in her expectation of life and having a child with disability may make it more challenging for the family, and more specifically for her. According to predictors of FQOL, the results of this study show that mothers’ variables (age and education) are more related to FQOL than fathers’ variables. This may be linked to the fact that in some Arab countries, it is still presumed that mothers are more responsible for caretaking, such as giving love and attention to their children, compared with fathers [35]. Additionally, the results reveal that marital status showed no significant effect. This lack of effect may be explained by the relative stereotypical gender roles and the role of extended families in Arab countries. The role that an extended family plays in countries like Saudi Arabia, where this extended family could compensate for the absence of one parent.

Another interesting finding of this study is that items within the disability-related support domain were the lowest for participants to express satisfaction compared to other domains in the scale. This shows that families must have more support in the form of services to overcome a family member’s disability. For the Turkish sample [6], satisfaction with the physical/material well-being domain was the lowest, while it was highest for this study sample. This is not surprising as it can solved by spending more money to cover the service, and Saudi Arabia’s authorities are providing material well-being to citizens. Contrarily, the American sample showed the lowest satisfaction with the emotional well-being domain [17].

It is important to note that the tool used in this study might not capture detailed information on family roles and responsibilities. This limitation restricts our understanding of family dynamics. Future research could include specific questions about who does what in the family to provide a clearer picture of family functioning. Such detailed data would enhance the comparability of studies and inform more targeted support interventions. In addition, this study primarily focused on the support currently provided to families, which was described in broad terms. To better understand the specific needs of families, future research could ask participants about the kinds of support they require. This information could reveal important differences between families and inform policy decisions to address any support shortfalls. Comparing the types of support available in different countries could provide additional context and insights.

### Implications for Research and Practice

The study data show that FQOL experience is rather low to moderate, indicating the need for intervention. Therefore, this study is consistent with Schmidt et al.’s (2017, [20] p. 87) conjecture that “a substantial amount of work has to be done in the future to provide appropriate and efficient support for families with children with disabilities”.

For future research, it would be interesting to conduct more research with the dependent sample where the individual quality of life of all family members and FQOL will be assessed from each part, specifically to investigate the link between the quality of life aspect within a family. However, in today’s world, it may not always be easy to understand who is a member of a family and maybe not solely relatives but also those people who are close to each other should be included (see, e.g., Brown & Brown, 2014 [13]). In addition, only 18% of this study sample were parents who were not living together. Therefore, future research must analyze a larger sample of divorced/separated parents and investigate in-depth the differences between these groups.

Future research can also examine the relationship between FQOL and faith, as well as the level of support. It is expected that adding faith to future research in Saudi Arabia to show the impact on FQOL. It is expected that people who express a high level of faith are likely to express more satisfaction with FQOL (even if not true) because being conservative in terms of faith is to believe that this is a kind of test that will make someone capable of coping with challenges in life to be rewarded in the afterlife. Combining qualitative and quantitative research would be useful to gain deeper insights into families’ responses and the underlying reasons.

## 5. Limitations

There are a few limitations to consider when interpreting the findings of this study. Firstly, its focus on the Riyadh region in Saudi Arabia may limit the generalizability of the results to other areas or cultural contexts. Secondly, its cross-sectional design captures only a specific moment in time, which prevents establishing causal relationships between the examined variables and family quality of life. Thirdly, the study’s scope excluded specific data on the ages of children with disabilities, a factor that might impact family quality of life. Inclusion of this variable is recommended in future research to enhance understanding of its potential effects on the dynamics within families of children with disabilities.

Moreover, while we identified the significant role of mothers in caregiving, this study did not delve deeply into the societal pressures and the cultural context. Future research could explore these cultural dimensions and their impact on FQOL more comprehensively. Qualitative studies focusing on mothers’ narratives could provide deeper insights into their experiences and the societal expectations that influence their quality of life.

Lastly, the study primarily employed descriptive analysis to explore FQOL trends. We acknowledge, as a limitation, the absence of direct statistical comparisons of FQOL means among different family members, which was influenced by the sample’s varied representation. Future research with a more balanced sample could facilitate these detailed comparisons. In addition, the absence of data on residential status could have provided additional insights into the FQOL. Future studies are encouraged to include this variable to further enhance our understanding of its impact on families of children with disabilities. In addition, it is important to note the absence of direct structural evidence supporting the extraction of a single composite score specifically for our sample. Future research should consider exploring the bi-factor or higher-order factor models to validate the composite score approach in different cultural contexts.

## 6. Conclusions

This study investigated the family quality of life (FQOL) of Saudi Arabian families with a member having an intellectual or developmental disability and identified the predictors that might significantly influence their FQOL. Our findings revealed several critical insights, including that families reported a moderate level of FQOL, indicating areas for improvement in support and resources. Key predictors of FQOL included the mother’s age and education, with younger mothers coping better and lower educational levels associated with higher FQOL, possibly due to differing expectations and societal pressures. The severity of the child’s disability also significantly impacted FQOL, with more severe disabilities correlating with lower satisfaction with FQOL. Consequently, it is essential for policymakers and service providers in Saudi Arabia to develop comprehensive support systems that involve mothers and cater to their specific needs. Future research could delve deeper into cultural and societal norms, possibly through qualitative studies that explore mothers’ narratives to gain a more nuanced understanding of their experiences and societal expectations. Longitudinal studies are also recommended to understand the evolving dynamics within these families over time and establish functional relationships between various predictors and FQOL. Additionally, examining the role of faith and community support in enhancing FQOL can inform more effective intervention strategies.

## Figures and Tables

**Table 1:** Means and standard deviations.

Variables		Mean	%	Std. Deviation
Type of disability	ID	3.32	48%	0.99
	Others	3.60	52%	0.93
Parents’ marital status	Parents are together	3.46	82%	0.95
	Parents are not together	3.48	18%	1.06
Mother’s education	Graduate studies	3.27	2%	1.00
	Bachelor	3.36	25%	0.97
	High school	3.47	24%	1.00
	Less than high school	3.71	27%	0.87
	Not educated *	3.66	22%	0.59
Father’s education	Graduate studies	3.62	4%	0.72
	Bachelor	3.41	36%	0.99
	High school	3.45	28%	0.90
	Less than high school	3.49	22%	1.06
	Not educated *	3.55	10%	0.99
Severity of the disability	Mild	3.67	33%	1.00
	Moderate	3.44	52%	0.95
	Severe	3.09	13%	0.89
Relationship with the PWD	Father	3.24	26%	0.99
	Mother	3.27	27%	1.07
	Brother	3.62	16%	0.88
	Sister	3.79	11%	1.01
	Others	3.75	20%	0.70
Overall		3.47		0.97

* Participants choose the option of “not educated” with no further information.

**Table 2:** Participants’ responses to the FQOL items.

		Responses					
Domain/Item		SS %	S %	N %	D %	SD %	Mean (Std. Deviation)
Family interaction 3.54 (1.17)							
1	My family enjoys spending time together	35	25	13	23	3	3.65 (1.26)
2	My family members talk openly with each other	30	27	15	21	6	3.53 (1.27)
3	My family solves problems together	30	25	17	20	8	3.50 (1.30)
4	My family members support each other to accomplish goals	34	26	9	23	7	3.56 (1.34)
5	My family members show that they love and care for each other	36	26	12	18	9	3.62 (1.35)
6	My family is able to handle life’s ups and downs	27	29	13	18	13	3.39 (1.37)
Parenting 3.44 (1.13)							
7	Family members help the children learn to be independent	31	26	13	22	8	3.49 (1.33)
8	Family members help children with schoolwork and activities	30	30	13	18	9	3.54 (1.33)
9	Family members teach children how to get along with others	34	25	11	20	9	3.54 (1.37)
10	Adults in my family teach children to make good decisions	31	27	12	20	10	3.47 (1.37)
11	Adults in my family know other people in their children’s lives (i.e., friends, teachers).	19	37	14	18	12	3.33 (1.28)
12	Adults in my family have time to take care of every child’s individual needs.	21	33	14	20	12	3.31 (1.31)
Emotional well-being 3.35 (1.08)							
13	My family has the support we need to relieve stress	23	32	16	18	11	3.38 (1.30)
14	My family members make friendship with others who provide support	19	30	20	20	11	3.26 (1.28)
15	My family members have some time to pursue their own interests	23	33	16	16	12	3.39 (1.32)
16	My family has outside help available to take care of the special needs of all family members.	24	28	21	15	11	3.38 (1.29)
Physical/material well-being 3.76 (1.02)							
17	My family members have transportation to take them to the places they must visit	38	31	13	12	6	3.82 (1.25)
18	My family gets dental care when needed	35	32	13	12	8	3.72 (1.27)
19	My family gets medical care when needed	37	36	10	12	5	3.89 (1.16)
20	My family has a way of taking care of our expenses	34	31	13	13	8	3.70 (1.28)
21	My family feels safe at home, work, school, and in our neighborhood	35	31	10	15	8	3.69 (1.31)
Disability-related support 3.15 (1.14)							
22	My family member with special needs has support to make progress at school or workplace	18	28	23	25	6	3.27 (1.19)
23	My family member with special needs has support to make progress at home.	21	25	18	26	10	3.22 (1.30)
24	My family member with special needs has support to make friends	21	23	18	26	13	3.13 (1.34)
25	My family has a good relationship with service providers who work with our family member with a disability	18	24	14	22	21	2.98 (1.42)

Note. SS = strongly satisfied, S = satisfied, N = Not sure, D = dissatisfied, SD = strongly dissatisfied.

**Table 3:** Regression results of FQOL.

Predictors	Unstandardized Coefficients		Standardized Coefficients	t	Sig.	Partial Correlation
	B	Std. Error	Beta			
(Constant)	3.539	0.479		7.383	0.00	
Type of disability (ID vs. others)	0.244	0.108	0.129	2.27	0.02	0.144
Severity of the disability	−0.289	0.082	−0.2	−3.545	0.00 *	−0.221
Monthly income	−0.132	0.064	−0.128	−2.063	0.04	-0.131
Father’s age	−0.022	0.011	−0.206	−1.966	0.05	−0.125
Mother’s age	0.059	0.012	0.499	4.796	0.00 *	0.293
Parents’ marital status	−0.259	0.175	−0.084	−1.48	0.14	−0.094
Father’s education	0.086	0.071	0.096	1.211	0.22	0.077
Mother’s education	−0.259	0.064	−0.308	−4.064	0.00 *	−0.252

R = 0.488, R^2^ = 0.238, *p* < 0.001; * = *p* is significant at level *p* < 0.01.

## Data Availability

The data presented in this study are available on request from the first author due to privacy.

## Appendix A. Scale Reliability Statistics

**Table:**

	Estimate	McDonald’s ω	Cronbach’s α
Family interaction	Point estimate	0.946	0.946
	95% CI lower bound	0.937	0.937
	95% CI upper bound	0.956	0.955
Parenting	Point estimate	0.924	0.923
	95% CI lower bound	0.911	0.909
	95% CI upper bound	0.937	0.936
Emotional well-being	Point estimate	0.856	0.854
	95% CI lower bound	0.831	0.825
	95% CI upper bound	0.882	0.878
Physical/material well-being	Point estimate	0.876	0.874
	95% CI lower bound	0.855	0.850
	95% CI upper bound	0.898	0.895
Disability-related support	Point estimate	0.895	0.891
	95% CI lower bound	0.876	0.870
	95% CI upper bound	0.914	0.909
Overall	Point estimate	0.966	0.965
	95% CI lower bound	0.960	0.960
	95% CI upper bound		

## Appendix B. Confirmatory Factor Analysis (Fit Indices)

**Table:**

Index	Value
Comparative Fit Index (CFI)	0.931
Tucker–Lewis Index (TLI)	0.922
Bentler–Bonett Non-normed Fit Index (NNFI)	0.922
Goodness of fit index (GFI)	0.904
Root mean square error of approximation (RMSEA)	0.074
RMSEA 90% CI lower bound	0.067
RMSEA 90% CI upper bound	0.080
